# Fluid Extract Phytolacca Decandra

**Published:** 1874-01

**Authors:** George A. Dyer

**Affiliations:** Washington, Indiana


Fluid Extract Phytolacca Decandra.—For some years past I have noticed in this country a strong disposition, especially in primiparæ, to mamary abscess, and in many cases have seen remedies fail to prevent the same, but, since reading Dr. Bigger's article on the use of this medicine in mastitis, I have, in five cases, used Tilden's fluid extract phytolacca in thirty drop doses every three hours, with the effect in every case of curing within thirty-six hours, and in one case where the lochia were suppressed of restoring the same. It acts like a charm.

George A. Dyer, M.D.

Washington, Indiana.

